# The Synergetic Effect Induced High Electrochemical Performance of CuO/Cu_2_O/Cu Nanocomposites as Lithium-Ion Battery Anodes

**DOI:** 10.3389/fchem.2021.790659

**Published:** 2021-11-22

**Authors:** Lin-Hui Wang, Shang Gao, Long-Long Ren, En-Long Zhou, Yu-Feng Qin

**Affiliations:** ^1^ College of Information Science and Engineering, Shandong Agricultural University, Taian, China; ^2^ School of Science, Shandong Jiaotong University, Jinan, China; ^3^ College of Mechanical and Electronic Engineering, Shandong Agricultural University, Taian, China; ^4^ College of Chemistry and Material Science, Shandong Agricultural University, Taian, China

**Keywords:** CuO/Cu_2_O/Cu nanocomposites, CuO nanowires, lithium-ion batteries, anodes, water bath method, electrochemical performance

## Abstract

Due to the high theoretical capability, copper-based oxides were widely investigated. A facile water bath method was used to synthesis CuO nanowires and CuO/Cu_2_O/Cu nanocomposites. Owing to the synergetic effect, the CuO/Cu_2_O/Cu nanocomposites exhibit superior electrochemical performance compared to the CuO nanowires. The initial discharge and charge capacities are 2,660.4 mAh/g and 2,107.8 mAh/g, and the reversible capacity is 1,265.7 mAh/g after 200 cycles at 200 mA/g. Moreover, the reversible capacity is 1,180 mAh/g at 800 mA/g and 1,750 mAh/g when back to 100 mA/g, indicating the excellent rate capability. The CuO/Cu_2_O/Cu nanocomposites also exhibit relatively high electric conductivity and lithium-ion diffusion coefficient, especially after cycling. For the energy storage mechanism, the capacitive controlled mechanism is predominance at the high scan rates, which is consistent with the excellent rate capability. The outstanding electrochemical performance of the CuO/Cu_2_O/Cu nanocomposites indicates the potential application of copper-based oxides nanomaterials in future lithium-ion batteries.

## Introduction

Rechargeable Lithium-ion batteries (LIBs) have been widely used in many fields, such as electric vehicles, cameras, and other portable electronic devices, because of their high working potential, lack of memory effect, and high energy density (Wang et al., 2020; Hu et al., 2020; Teng et al., 2020; Zhang et al., 2020; Zuo and Gong, 2020; Li et al., 2021; Wang et al., 2021; Li et al., 2021; Li et al., 2021d; Wu et al., 2021; Liang et al., 2022). However, the traditional graphite anodes cannot meet further demands of high-power hybrid electric vehicles in the future because of the relatively inferior rate performance and the low theoretical capacity (372 mAh/g) (Shen et al., 2013; Zhang et al., 2013; Zhou et al., 2014; Wang et al., 2020; Pan et al., 2020; Wang et al., 2021). The transition metal oxides have been widely researched for their low cost, widespread availability, and high theoretical capacities (500–1,000 mAh/g) (Shen et al., 2013; Zhang et al., 2013; Zhou et al., 2014; Ananya et al., 2016; Pan et al., 2020). Especially, copper-based oxides were paid much more attention owing to their abundance, environment-friendly, superior rate performance, and high theoretical capacities (674 mAh/g for CuO and 375 mAh/g for Cu_2_O) (Shen et al., 2013; Zhang et al., 2013; Wang et al., 2014; Zhou et al., 2014; Ananya et al., 2016; Kim et al., 2019; Pan et al., 2020; Zhang et al., 2021). However, there are also some intrinsic disadvantages, such as volume expansion and low electric conductivity during the charge-discharge process, which should be resolved (Shen et al., 2013; Zhang et al., 2013; Zhou et al., 2014; Ananya et al., 2016; Pan et al., 2020). To solve these problems, many different kinds of nanostructures and morphologies were designed and prepared by many kinds of methods (Wang et al., 2014; Wang et al., 2014; Xu et al., 2016; Kim et al., 2019; Yuan et al., 2019; Zhang et al., 2021). Zhang et al. (2013) synthesized porous CuO nanosphere film which was used as anodes, exhibiting a high reversible discharge capacity of 799.7 mAh/g. Park et al. prepared structure-controlled octahedral Cu_2_O nanostructures using polymers as additives during the synthesis, which exhibited superior electrochemical performance (Kim et al., 2019). The CuO nanoparticles with different diameters were also prepared to clarify the effects of the particle’s size, delivering a reversible capacity of 540 mAh/g (Wang et al., 2014). Moreover, carbon-based materials were added to enhance the electric conductivity and to relieve the pulverization of the copper-based oxides (Huang et al., 2011; Mai et al., 2011; Shen et al., 2013; Zhou et al., 2014; Chen et al., 2015; Xu et al., 2015; Ananya et al., 2016; Kim et al., 2019; Pan et al., 2020; Trukawka et al., 2021; Zhang et al., 2021). Mu et al. reported that the Cu_2_O nanoparticles distributed in the porous carbon channels exhibited a high capacity of 884.4 mAh/g (Zhou et al., 2014). Tu et al. synthesized CuO/graphene composite, whose electrochemical performance was improved with a discharge capacity of 583.5 mAh/g (Mai et al., 2011). Cu_2_O@GO composite with a core-shell structure was also synthesized, exhibiting a discharge capacity of 458 mAh/g (Xu et al., 2015).

Recently, due to the synergetic effect, many ternary and quaternary materials were investigated and exhibited excellent electrochemical performance (Shi et al., 2016; Yuan et al., 2017; Wu et al., 2018; Xu et al., 2019; Wang et al., 2020; Gao et al., 2020; Murphin Kumar et al., 2020; Wang et al., 2021b; Li et al., 2021c; Trukawka et al., 2021). Wang et al. prepared Co_3_O_4_/CuO composite, which exhibited a high charge-discharge capacity of 1,056 mAh/g (Shi et al., 2016). Krishnan et al. fabricated porous Cu_2_O: Mo microspheres, which showed a reversible capacity of 1,082 mAh/g (Murphin Kumar et al., 2020). Gao et al. synthesized polypyrrole coated Cu/Cu_2_O nanowire, which exhibited an increased capacity of 787 mAh/g (Wang et al., 2020b). Tang et al. prepared C@SnO_2_/Cu_2_O nanosheet clusters, which exhibited an initial discharge capacity of 1726 mAh/g at 0.5C (Wang et al., 2021b). The synergetic effect of more than three components is crucial to develop the electrochemical performance of copper-based oxides. However, the specific capacity and rate performance should be further improved to satisfy the demands of future applications.

In this work, CuO nanowires and CuO/Cu_2_O/Cu nanocomposites were prepared by a facile water bath method that has not been used to prepare copper-based oxides anodes before. The initial discharge capability and charge capacity of CuO nanowires are 1,061.8 mAh/g and 922.1 mAh/g at 200 mA/g. While for the CuO/Cu_2_O/Cu nanocomposites, they are 2,660.4 mAh/g and 2,107.8 mAh/g with a high initial Coulombic efficiency of 79.23%. The Coulombic efficiency increases to 98% in the second cycle and is maintained near 100% in the following cycles. The reversible capacity of 1,265.7 mAh/g was obtained after 200 cycles. Moreover, the reversible capacity is 1,180 mAh/g at 800 mA/g and increases to 1,750 mA/g when back to 100 mA/g, which indicates the excellent rate performance. As far as we know, the CuO/Cu_2_O/Cu nanocomposites exhibit superior electrochemical performance to the reported results of copper-based oxides, which could be due to the synergetic effect and the facile water bath method. Our results indicate that the copper-based oxides anodes with a synergetic effect will satisfy the demands of the next-generation LIBs.

## Experimental Section

### Materials and Methods

**FIGURE 1:**
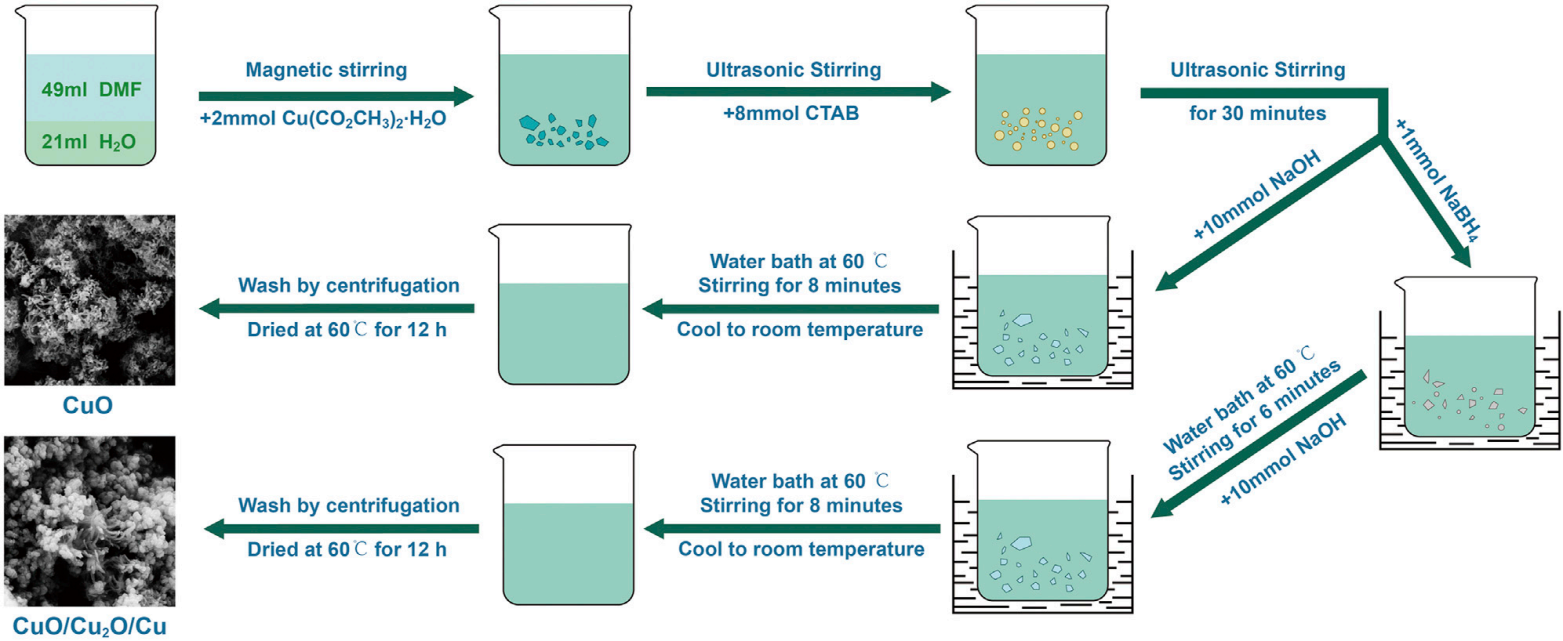
The schematic illustration of preparing CuO nanowires and CuO/Cu_2_O/Cu nanocomposites.

The schematic illustration of preparing CuO nanowires and CuO/Cu_2_O/Cu nanocomposites was shown in Figure 1. The mixed solution of 49 ml dimethylformamide (DMF) and 21 ml pure water was magnetically stirred for 2 min in a beaker, and then 2 mmol copper acetate monohydrate Cu(CO_2_CH_3_)_2_·H_2_O was added. After ultrasonic stirring for 30 min to thoroughly dissolve, 8 mmol hexadecyl trimethyl ammonium bromide (CTAB) was added successively. After another 30 min of ultrasonic stirring, 10 mmol NaOH was added. Then the above solution was put into a 60°C water bath and magnetically stirred for 8 min. When naturally cooled to room temperature, the solution was centrifuged by pure water and ethanol, in turn, many times. The obtained black precipitates were dried at 60°C for 12 h in a vacuum oven, and finally, the CuO nanowires were obtained. The preparing route of the CuO/Cu_2_O/Cu nanocomposites was the same as the above process, except that 1 mmol of reductive agent NaBH_4_ was added and stirred for 6 min before adding 10 mmol NaOH.

### Structure and Morphology

The structure and morphology of the CuO nanowires and CuO/Cu_2_O/Cu nanocomposites were characterized by X-ray diffraction (XRD, Smart Lab, Rigku Japan) and scanning electron microscope (SEM, GeminiSEM300, Zeiss, Germany). The XRD measurements were performed in the range of 20°–80° at a scan rate of 3°/min using a Cu Кα radiation.

### Electrochemical Performance Characterization

The testing anodes were mixed with as-prepared active materials (CuO nanowires or CuO/Cu_2_O/Cu nanocomposites, 70wt%), carbon black (20wt%), and carboxymethyl cellulose (CMC, 10wt%) dissolved in pure water. The black slurry was uniformly coated on a copper foil and dried at 60°C for 12 h in a vacuum oven. Finally, the obtained coated foil was punched into disks with a diameter of 12 mm. The half-cells (CR-2032) were assembled in a glove box. Lithium metal disk and the Celgard 2,250 film were used as the counter electrode and the diaphragm, respectively. The electrolyte was 1M LiPF6 dissolved in a mixture of ethyl carbonate (EC, 50 v/v%) and dimethyl ethyl carbonate (DEC, 50 v/v%). The electrochemical performance was measured by battery measuring systems (Land-ct2001A, China) and electrochemical workstation (CHI660E, China) with potential from 0.01 to 3.0 V at room temperature. To make sure the thorough penetration of the electrolyte, all cells were left to stand for 12 h before being measured.

## Results and Discussion

### Structure and Morphology

**FIGURE 2:**
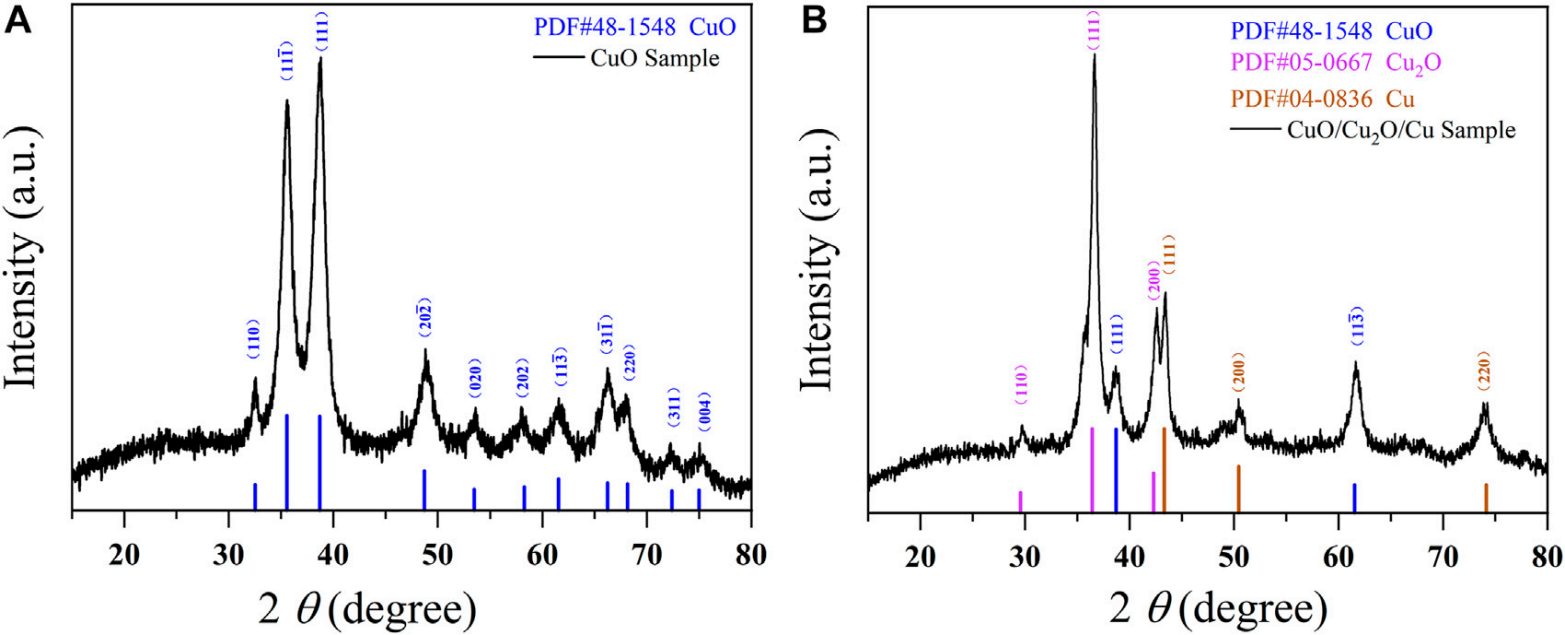
The XRD patterns of CuO nanowires **(A)** and CuO/Cu_2_O/Cu nanocomposites **(B)**.

As is shown in Figure 2A, the XRD patterns of CuO nanowires consist with the standard card of PDF No. 48–1548 (CuO) with no other diffraction peak, which indicates the purity of CuO nanowires. The characteristic peaks at 32.5°, 35.5°, 38.7°, 48.7°, 53.5°, 58.3°, 61.5°, 66.2°, 68.1°, 72.4°, and 75.0° stand for (110), (11 $\bar{1}$ ), (111), (20 $\bar{2}$ ), (020), (202), (11 $\bar{3}$ ), (31 $\bar{1}$ ), (220), (311), and (004) crystal plane of monolithic CuO phase respectively, which indicates the good crystallinity of CuO (Huang et al., 2011; Chen et al., 2015; Shi et al., 2016; Yuan et al., 2017; Wu et al., 2018; Xu et al., 2019; Murphin Kumar et al., 2020; Li et al., 2021c; Trukawka et al., 2021). From the XRD patterns of CuO/Cu_2_O/Cu nanocomposites shown in Figure 2B, we can see that the as-prepared sample comprises CuO, Cu_2_O, and Cu phases. The characteristic peaks at 38.7° and 61.5° signify (111) and (11 $\bar{3}$ ) crystal plane of monolithic CuO phase (PDF No. 48–1548) (Huang et al., 2011; Chen et al., 2015; Shi et al., 2016; Yuan et al., 2017; Wu et al., 2018; Xu et al., 2019; Trukawka et al., 2021), and the peaks at 29.6°, 36.4°, and 42.3° index to (110), (111), and (200) crystal plane of cubic Cu_2_O phase (PDF No. 05–0667) (Wu et al., 2018; Xu et al., 2019; Wang et al., 2020b; Gao et al., 2020; Murphin Kumar et al., 2020; Wang et al., 2021b; Li et al., 2021c), and the peaks of 43.3°, 50.4°, and 74.1° assign to (111), (200), and (220) crystal plane of cubic Cu phase (PDF No. 04–0836) (Wang et al., 2020b; Wang et al., 2021b; Li et al., 2021c). The appearance of Cu_2_O and Cu phases was caused by the reductive reaction induced by the common mild reductive agent of NaBH_4_. The weight ratios of CuO, Cu_2_O, and Cu phases are 17.8, 60.5, and 21.7%, respectively, which was calculated by the intensity ratio of the 111) crystal plane peaks ( $I_{\text{CuO }}:I_{{\text{Cu}}_{2}\text{O }}:I_{\text{Cu }}$ ) (Yu et al., 2007; Hu et al., 2013; Zhou et al., 2014; Meng et al., 2015; Kim et al., 2016; Xu et al., 2019; Li et al., 2021c).

**FIGURE 3:**
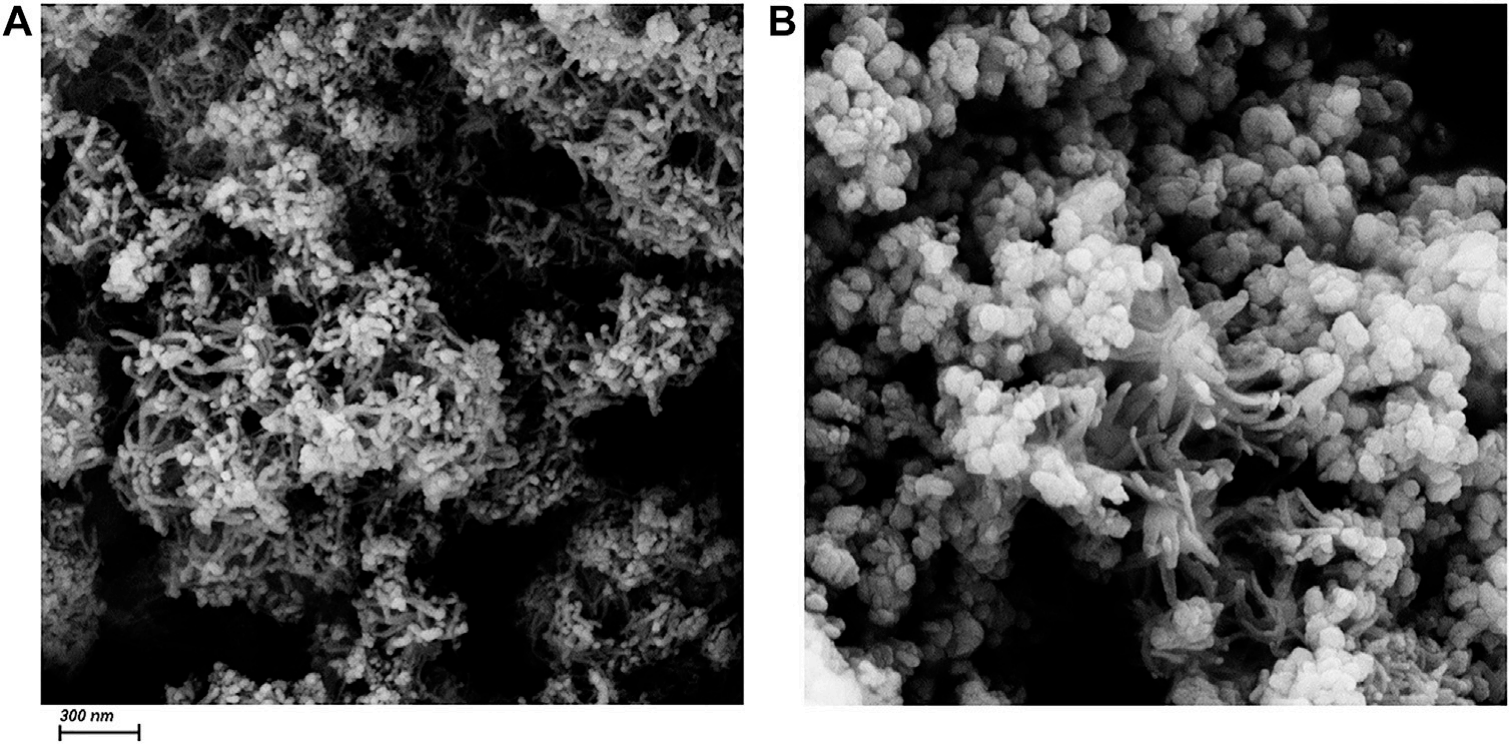
The SEM images of CuO nanowires **(A)** and CuO/Cu_2_O/Cu nanocomposites **(B)**. The scale bar is 300 nm.

As is shown in Figure 3, the morphology of CuO nanowires and CuO/Cu_2_O/Cu nanocomposites can be identified by the SEM images with a scale bar of 300 nm. From Figure 3A, we can see a lot of tangled nanowires with an average diameter of about 25 nm. But we can’t obtain the length of these nanowires because of the tangled morphology. CuO/Cu_2_O/Cu nanocomposites consist of a part of nanowires and much more nanoparticles with an average diameter of 95 nm, as is shown in Figure 3B. The appearance of the nanoparticles in the SEM image consists with the appearance of Cu_2_O and Cu phases in the XRD pattern due to the reductive reaction. Therefore, it is reasonable to conclude that the nanowires are the CuO phase not reduced, and the nanoparticles should be the Cu_2_O or Cu phases. The small number of nanowires in the SEM image is also consistent with the small amount of CuO (17.8%) estimated by the XRD patterns.

### Electrochemical Performance

**FIGURE 4:**
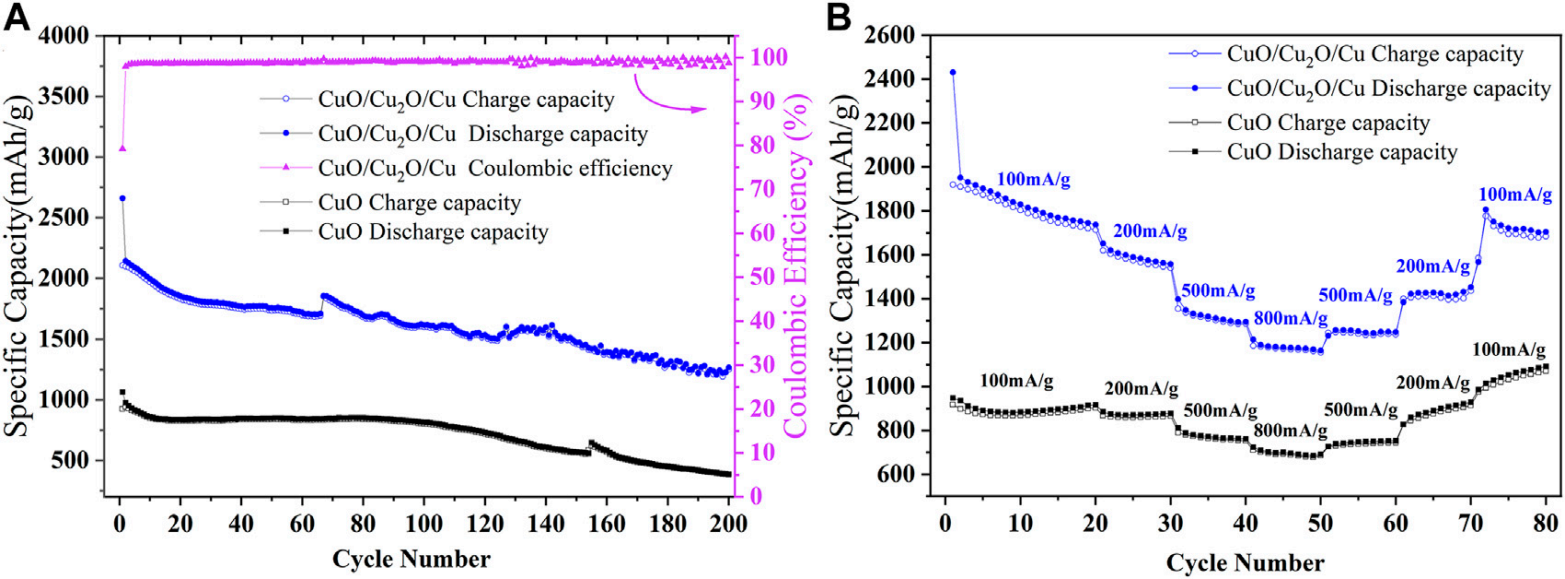
The cycle stability **(A)** at a current density of 200 mA/g and rate capability **(B)** at different current densities of CuO nanowires and CuO/Cu_2_O/Cu nanocomposites.

The cycle stability at 200 mA/g and the rate capability of CuO nanowires and CuO/Cu_2_O/Cu nanocomposites are shown in Figure 4. From the cycle curves shown in Figure 4A, the initial discharge capacity and charge capacity of CuO nanowires are 1,061.8 mAh/g and 922.1 mAh/g, respectively. which is better than many previous reports of copper-based oxides (Shen et al., 2013; Zhang et al., 2013; Wang et al., 2014a; Wang et al., 2014b; Zhou et al., 2014; Ananya et al., 2016; Xu et al., 2016; Kim et al., 2019; Yuan et al., 2019; Pan et al., 2020; Zhang et al., 2021). However, the reversible capability decreases to 385 mAh/g after 200 cycles. While for the CuO/Cu_2_O/Cu nanocomposites, the initial discharge capacity and charge capacity were 2,660.4 mAh/g and 2,107.8 mAh/g with an initial Coulombic efficiency of 79.23%. The Coulombic efficiency increased to 98% in the second cycle and was maintained near 100% in the following cycles. Furthermore, the reversible capacity of 1,265.7 mAh/g after 200 cycles was obtained, which is the highest of all reported results as far as we know (Huang et al., 2011; Mai et al., 2011; Shen et al., 2013; Zhang et al., 2013; Wang et al., 2014a; Wang et al., 2014b; Zhou et al., 2014; Chen et al., 2015; Xu et al., 2015; Ananya et al., 2016; Shi et al., 2016; Xu et al., 2016; Yuan et al., 2017; Wu et al., 2018; Kim et al., 2019; Xu et al., 2019; Yuan et al., 2019; Pan et al., 2020; Trukawka et al., 2021; Zhang et al., 2021). The jumps in the cycle curves at the 155th and 65th cycles for CuO nanowires and CuO/Cu_2_O/Cu nanocomposites were caused by the interruption of electrical power.

To test the rate performance, the charge-discharge cycles of our samples at different current densities were carried out, as is shown in Figure 4B. The reversible capabilities of CuO nanowires are 885, 870, 772, and 704 mAh/g at 100, 200, 500, and 800 mA/g, and the capabilities increase to 747, 890, and 1,052 mAh/g when back to 500, 200, and 100 mA/g, which indicates the excellent rate performance because of the unique tangled nanowires structure and the water bath method. The reversible capabilities of CuO/Cu_2_O/Cu nanocomposites are 1,840, 1,590, 1,319, and 1,180 mAh/g at 100, 200, 500, and 800 mA/g, and the capabilities increase to 1,252, 1,428, and 1750 mAh/g when back to 500, 200, and 100 mA/g, which is much better than that of CuO nanowires. The electrochemical results of CuO nanowires and CuO/Cu_2_O/Cu nanocomposites in this work and some previously reported results of copper-based oxides were listed in Table 1. To our knowledge, the electrochemical performance of the CuO/Cu_2_O/Cu nanocomposites was superior to all the reported results of copper-based oxides, which could be due to the synergetic effect and the water bath method. In our case, the Cu composite may provide the conductive network and the accommodation of the volume explanation during the cycles to improve the cycling stability, and the CuO and Cu_2_O composites may provide more active sites to enhance the reversible capacity.

**TABLE 1:** The list of electrochemical results in this work and some reported results of copper-based oxides.

**Materials**	**Initial discharge capacity (mAh/g)**	**Initial coulombic efficiency**	**Reversible capacity (mAh/g)**	**Current density (mA/g)**	**References**
CuO/Cu_2_O/Cu	2,660.4	79.23%	1,265.7 (200 cycles)	200	This work
CuO	1,061.8	86.84%	385 (200 cycles)	200	This work
CuO-Cu_2_O	1,083	65.1%	487 (60 cycles)	200	Zhou et al. (2014)
CuO	803.7	70.4%	799.7 (100 cycles)	0.1C	Zhang et al. (2013)
Cu_2_O	1,383.9	64%	884.4 (100 cycles)	100	Shen et al. (2013)
Cu_2_O-Mn_3_O_4_	1,257	72.3%	792 (350 cycles)	1,500	Pan et al. (2020)
Cu_2_O/HEG	2050 (0.05C)	50%	430 (25 cycles)	0.1C	Ananya et al. (2016)
Cu_2_O	1,100	36.4%	666 (200 cycles)	200	Kim et al. (2019)
CuO/Cu_2_O/Coal	1,200	65%	767 (200 cycles)	100	Zhang et al. (2021)
CuO	1,000	56.2%	677.1 (50 cycles)	0.1C	Wang et al. (2014a)
Cu@CuO	672.7	67.7%	354.1 (200 cycles)	100	Yuan et al. (2019)
CuO	1,196	56.2%	540 (100 cycles)	0.5C	Wang et al. (2014b)
CuO/graphene	817	68.7%	583.5 (50 cycles)	0.1C	Mai et al. (2011)
Cu_2_O	1,097	65.8%	458 (50 cycles)	100	Xu et al. (2015)
CuO/C	1,150	50%	470 (50 cycles)	100	Huang et al. (2011)
Co_3_O_4_/CuO	1,229	75.8%	1,056 (500 cycles)	200	Shi et al. (2016)
Cu_2_O/CuO/rGO	600	-	550 (100 cycles)	0.5C	Wu et al. (2018)
CuO/Cu_2_O/C	887 (20 mA/g)	44%	260 (600 cycles)	200	Xu et al. (2019)
Cu_2_O:Mo	1,128	-	1,082 (100 cycles)	100	Murphin Kumar et al. (2020)
Cu_2_O/CuO/Cu	508.9	55.8%	479.9 (150 cycles)	50	Li et al. (2021c)
Cu/Cu_2_O@Ppy	699.2	64.6%	787 (250 cycles)	100	Wang et al. (2020b)
C@/SnO_2_/Cu_2_O	1726	46.3%	814 (300 cycles)	0.5C	Wang et al. (2021b)
CuO/Cu_2_O	727	70.6%	740 (250 cycles)	100	Hu et al. (2013)
CuO	1,095 (100 mA/g)	59.5%	613.9 (100 cycles)	500	Li et al. (2018)
Cu_2_O	555	48%	559 (50 cycles)	2000	Liu et al. (2021)

**FIGURE 5:**
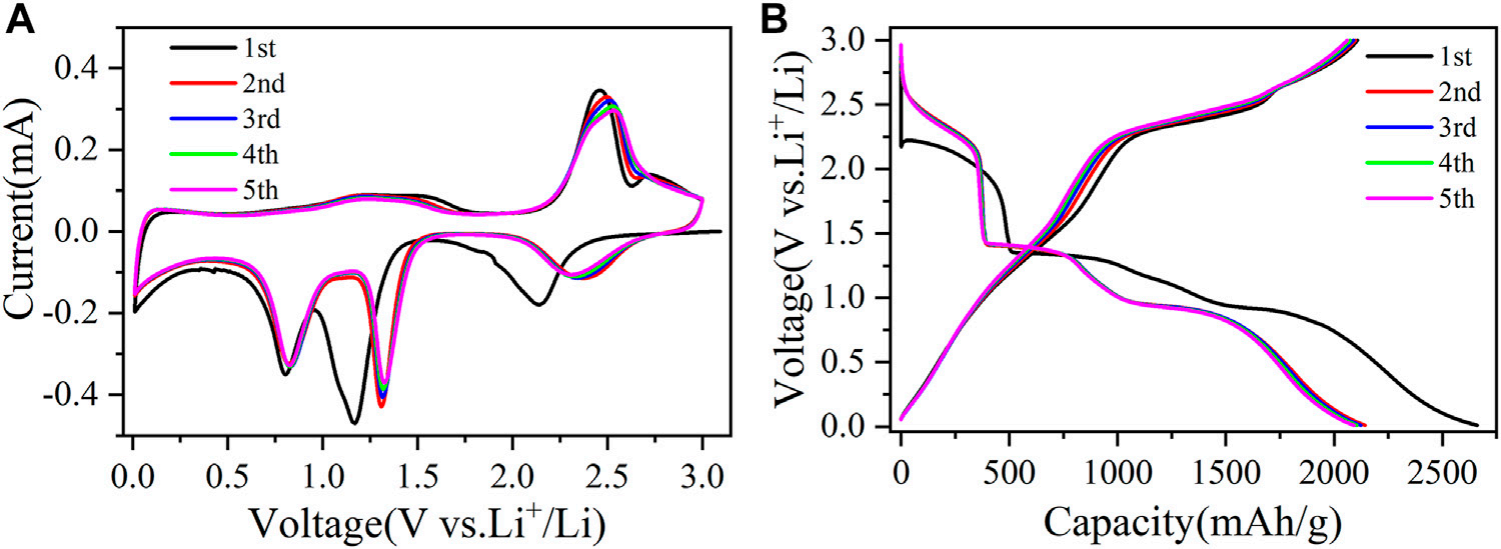
The first five cyclic voltammetry curves **(A)** at a scan rate of 0.1 mV/s and the first five charge-discharge curves **(B)** at a current density of 200 mA/g for CuO/Cu_2_O/Cu nanocomposites.

The first five cyclic voltammetry (CV) curves of CuO/Cu_2_O/Cu nanocomposites at a scan rate of 0.1 mV/s were shown in Figure 5A to clarify the electrochemical reaction mechanism. In the first cycle, there are three reduction peaks at 2.14, 1.17, and 0.8 V, which correspond to the reductive process of CuO to a ${\text{Cu}}_{1-x}^{\text{II}}{\text{Cu}}_{x}^{\text{I}}{\text{O}}_{1-x\text{/}2}\;\left(\text{0}\leq x\leq \text{0}\text{.}4\right)$ solid-solution mixed-phase (Huang et al., 2011; Yuan et al., 2019; Zhang et al., 2021), formation of Cu_2_O phase (Zhang et al., 2013; Wu et al., 2018; Zhang et al., 2021), and the following transformation into Cu phase as well as the formation of solid electrolyte interface (SEI) (Shi et al., 2016; Yuan et al., 2017; Li et al., 2021c). The three reduction peaks increase to 2.34, 1.32, and 0.83 V and overlap in the following cycles (Xu et al., 2019), which indicates the excellent reversible cycle stability (Huang et al., 2011; Chen et al., 2015; Shi et al., 2016; Yuan et al., 2017; Trukawka et al., 2021). There are also three oxidation peaks located at 1.57, 2.46, and 2.72 V in the first cycle, which were induced by the decomposition of the SEI layer (Shen et al., 2013; Wang et al., 2020b), the oxidation of Cu to Cu_2_O (Huang et al., 2011; Wang et al., 2014a; Yuan et al., 2019), and the further oxidation reaction to CuO (Huang et al., 2011; Yuan et al., 2019). In the second cycle, the oxidation peak at 1.57 V disappears due to the stability of the SEI layer. The peak at 2.72 V decreases to 2.70 V in the second cycle and then vanishes in the following cycles because of the polarization during electrode reaction (Wang et al., 2020b; Zhang et al., 2021). The position of oxidation peak at 2.46 V increases a little in the following four cycles due to the tiny structure change of copper-based oxides (Wang et al., 2014a; Xu et al., 2019).

The first five charge-discharge curves of CuO/Cu_2_O/Cu nanocomposites at 200 mA/g were also shown in Figure 5B to verify the results of CV curves. There are three discharge plateaus around 2.20–1.85, 1.35–1.10 V, and 0.95–0.75 V in the first discharge process, which consist with the reduction peaks in the initial CV cathodic sweep. In the first charge curve, the three charge platforms around 1.10–1.75, 2.20–2.52, and 2.60–2.84 V correspond to the oxidation peaks in the initial CV anodic sweep. The plateaus in the following discharge-charge cycles are consistent with the redox reactions peaks in the CV curves. Furthermore, the discharge-charge curves also almost overlap, indicating the reversible electrochemical reactions and the high reversible cycle capability (Yuan et al., 2017; Yuan et al., 2019; Murphin Kumar et al., 2020).

**FIGURE 6:**
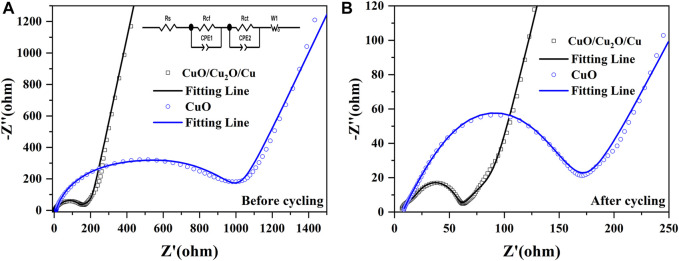
The electrochemical impedance spectroscopy of CuO/Cu_2_O/Cu nanocomposites and CuO nanowires before **(A)** and after **(B)** cycling between 10^–2^ Hz and 10^5^ Hz. The inset of **(A)** is the equivalent circuit.

As is shown in Figure 6, The electrochemical impedance spectroscopy (EIS) of CuO/Cu_2_O/Cu nanocomposites and CuO nanowires were measured between 10^–2^ Hz and 10^5^ Hz before and after cycling to further understand the reaction kinetics and the enhanced electrochemical performance (Yuan et al., 2017; Yuan et al., 2019; Wang et al., 2021b). Two semicircles and a straight line were found in the Nyquist plots (scatters) (Murphin Kumar et al., 2020; Wang et al., 2020b; Li et al., 2021c; Wang et al., 2021b), which are well fitted by the equivalent circuit (fitting lines). The equivalent circuit is shown in Figure 6A inset, and the parameters of *R*_s_ represents the ohmic resistance, *R*_cf_ signifies the impedance of the SEI layer, *R*_ct_ denotes the charge transfer resistance, *W*_1_ designates the Warburg impedance (Wang t al., 2020b; Murphin Kumar et al., 2020; Wang et al., 2021b; Li et al., 2021c). Moreover, the Li-ions diffusion coefficient ( $D_{{\text{Li}}^{+}}$ ) can be calculated by the equations below according to the EIS data in the low-frequency region (Shen et al., 2013; Zhou et al., 2014; Wang et al., 2020a; Gao et al., 2020).

$$D_{L{\text{i}}^{+}}=\frac{R^{2}T^{2}}{2A^{2}n^{4}F^{4}C^{2}\sigma^{2}}$$ (1)

$$Z_{\text{real}}=R_{\text{total}}+\sigma \omega^{-1/2}$$ (2)

The physical quantities of *R*, *T*, *A*, *n*, *F*, *C*, and *σ* denote the gas constant, the measuring temperature, the surface area of the electrode, the number of transferred electrons, the Faraday constant, the concentration of lithium ions, and the Warburg coefficient, respectively (Shen et al., 2013; Zhou et al., 2014; Wang et al., 2020a). The value of *σ* could be fitted by Eq. 2, in which the parameter of *ω* is the angular frequency (Gao et al., 2020). The fitted resistance parameters of EIS and the Li-ions diffusion coefficients before and after cycling are listed in Table 2.

**TABLE 2:** The fitted resistance parameters of electrochemical impedance spectroscopy and the Li-ions diffusion coefficients before and after cycling.

**Materials**	***R*_s_ (Ω)**	***R*_cf_ (Ω)**	***R*_ct_ (Ω)**	***R*_total_ (Ω)**	** $D_{{\text{Li}}^{+}}$ (cm^2^/s)**
CuO/Cu_2_O/Cu Before cycling	2.41	16.75	101	120.16	1.20 × 10^−15^
CuO/Cu_2_O/Cu After cycling	6.03	9.21	41.78	57.02	4.34 × 10^−12^
CuO nanowires Before cycling	10.49	389.3	94.87	494.66	4.82 × 10^−15^
CuO nanowires Before cycling	7.27	111.7	52.56	171.53	2.17 × 10^−13^

The *R*_cf_, *R*_ct_, and the total resistance of *R*_total_ for both CuO/Cu_2_O/Cu nanocomposites and CuO nanowires are much smaller after cycling, which indicates the increased electric conductivity due to the structure change and the formation of Cu component during the cycling process (Li et al., 2018; Hong et al., 2020; Wang et al., 2020b; Liu et al., 2021). The relatively high *R*_total_ (117.53 Ω) and the low Li-ions diffusion coefficient (2.17 × 10^−13^ cm^2^/s)of the CuO nanowires after cycling imply the low reversible capability of 385 mAh/g after 200 cycles shown in Figure 4. However, the CuO/Cu_2_O/Cu nanocomposites after cycling exhibit the lowest *R*_cf_ (9.21 Ω), *R*_ct_ (41.78 Ω), and *R*_total_ (57.02 Ω), as well as the highest Li-ions diffusion coefficient (4.34 × 10^−12^ cm^2^/s), indicating the higher electrochemical kinetics compared to the CuO nanowires, which are consistent with the outstanding electrochemical performance shown in Figures 4, 5 (Li et al., 2018; Hong et al., 2020; Liu et al., 2021). The outstanding electrochemical performance of the CuO/Cu_2_O/Cu nanocomposites could result from the unique nanocomposites structure and the synergetic effect of the components (Hu et al., 2013; Kim et al., 2016; Wang et al., 2020b; Murphin Kumar et al., 2020; Wang et al., 2021b; Li et al., 2021c).

**FIGURE 7:**
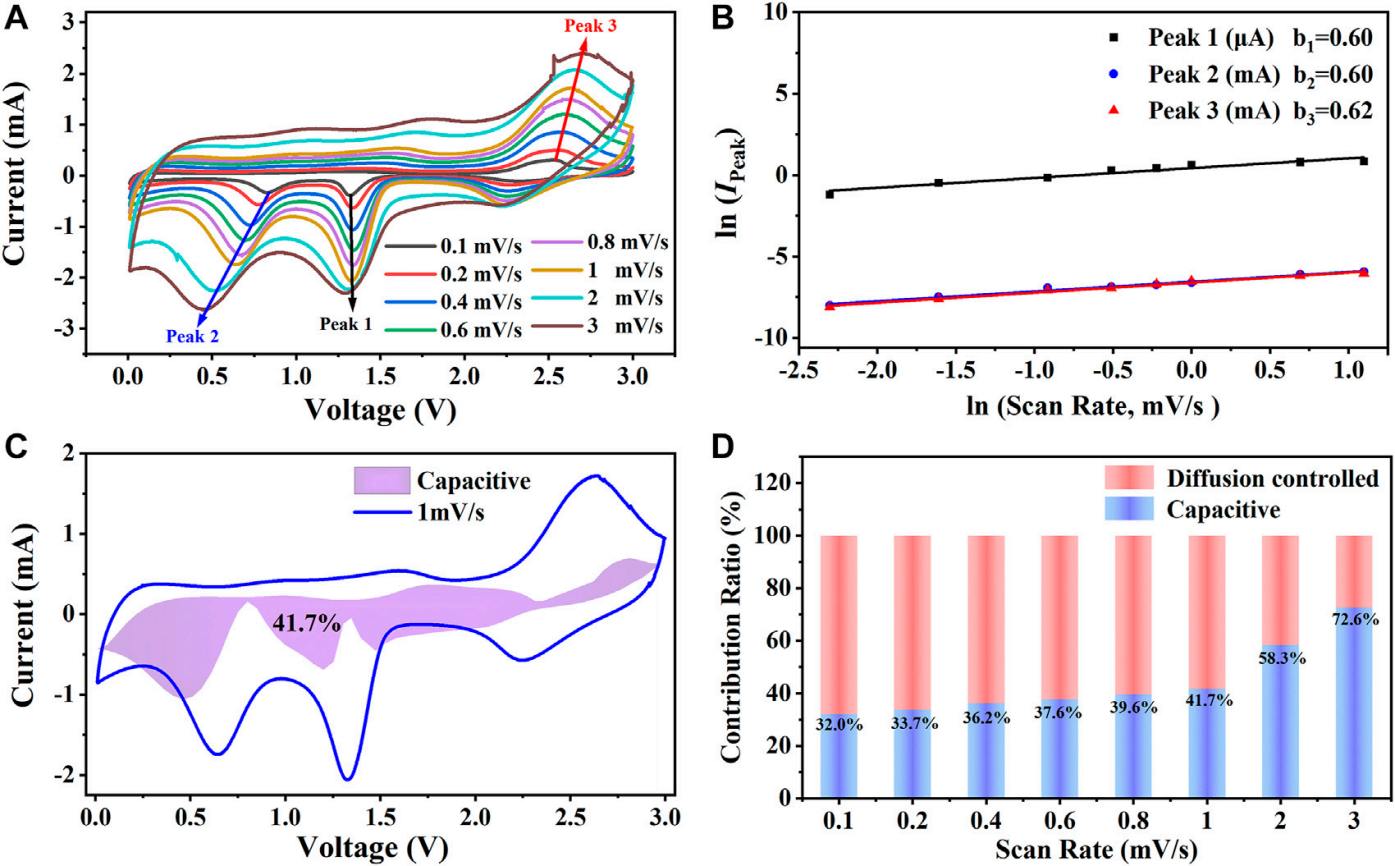
**(A)** The cyclic voltammetry curves of CuO/Cu_2_O/Cu nanocomposites with different scan rates. **(B)** The linear fitting results of log (*I*_Peak_) vs. log(*ν*). **(C)** Capacitive contribution at 1 mV/s. **(D)** Capacitive contributions to the lithium-ion storage at different scan rates.

On account of the outstanding electrochemical performance of the CuO/Cu_2_O/Cu nanocomposites, as is shown in Figure 7A, the CV curves with scan rates in the range of 0.1 mV/s—3 mV/s were further measured to clarify the energy storage mechanism. The shapes of the CV curves are much similar, and the redox peaks are obvious even at 3 mV/s, indicating the outstanding lithium-ion intercalation dynamics (Wang et al., 2020b). The positions and the values of the three redox peaks marked by the arrows in Figure 7A change regularly with the scan rates due to different reaction processes according to scan rates (Wang et al., 2020b; Liu et al., 2021; Zhang et al., 2021). The peak current (*I*_Peak_) can be expressed by the equations with scan rates (*ν*).

$$I_{\text{Peak}}\mathrm{=a}\;v^{b}$$ (3)

$$\text{ln}\left(I_{\text{Peak}}\right)=\;b\text{ ln}\left(v\right)\;+\text{ ln}a$$ (4)

The lithium-ion storage mechanism can be identified by the value of *b*, which is between 0.5 and 1 depending on the contribution ratios of diffusion-controlled (*b* = 0.5) and capacitance controlled (*b* = 1) (Li et al., 2018; Hong et al., 2020; Liu et al., 2021). According to Figure 7B, the values of *b* for the two reduction peaks and the oxidation peak are 0.6 and 0.62, respectively, indicating the diffusion-controlled mechanism is dominant (Li et al., 2018; Hong et al., 2020; Liu et al., 2021). The quantitative contribution ratios can be calculated by the equations below.

$$I=k_{1}v\;+\;k_{2}v^{\text{0}\text{.}5}$$ (5)

$$\mathrm{I/}v^{\text{0}\text{.}5}=k_{1}v^{\text{0}\text{.}5}+k_{2}$$ (6)

The parameters of *k*_1_ *ν* and *k*_2_ *ν* ^0.5^ represent the contribution of capacitance controlled and diffusion-controlled, respectively. *k*_1_ can be obtained by linear fitting of *I/ν* ^0.5^ vs. *ν* ^0.5^ in Eq. 6 at a certain voltage with different currents and scan rates. As is shown in Figure 7C, after obtaining enough numbers of *k*_1_, the capacitance contribution ratio was calculated by the area ratio of the shadow and the CV curves, and the ratio is 41.7% at 1 mV/s. As is shown in Figure 7D, at the low scan rates, the diffusion-controlled mechanism is dominant, while at the high scan rates, the capacitive controlled mechanism is predominance. The capacitance contribution ratio to the lithium-ion storage is up to 72.6% at the scan rate of 3 mV/s, which is consistent with the excellent rate capability shown in Figure 4B.

## Conclusion

In this work, CuO/Cu_2_O/Cu nanocomposites and CuO nanowires were prepared by a facile water bath method. The CuO nanowires exhibit a high initial discharge capacity and charge capacity of 1,061.8 mAh/g and 922.1 mAh/g at 200 mA/g, but a low reversible capability of 385 mAh/g after 200 cycles. While for the CuO/Cu_2_O/Cu nanocomposites, the initial capacities are 2,660.4 mAh/g and 2,107.8 mAh/g, respectively, and the reversible capacity is 1,265.7 mAh/g. The rate performance of the CuO/Cu_2_O/Cu nanocomposites was also excellent with a reversible capacity of 1,180 mAh/g at 800 mA/g and 1750 mA/g when back to 100 mA/g. The outstanding electrochemical performance of the CuO/Cu_2_O/Cu nanocomposites could result from the synergetic effect of multivalent states metal and the facile water bath method. Copper-based oxides nanomaterials with the synergetic effect could be used as anodes for lithium-ion batteries.

## Data Availability

The original contributions presented in the study are included in the article/Supplementary Material, further inquiries can be directed to the corresponding author.
